# *ZmDREB2.9* Gene in Maize (*Zea mays* L.): Genome-Wide Identification, Characterization, Expression, and Stress Response

**DOI:** 10.3390/plants11223060

**Published:** 2022-11-11

**Authors:** Mikhail A. Filyushin, Elena Z. Kochieva, Anna V. Shchennikova

**Affiliations:** Research Center of Biotechnology, Institute of Bioengineering, Russian Academy of Sciences, Leninsky Ave. 33, Bld. 2, Moscow 119071, Russia

**Keywords:** *Zea mays* L., DREB proteins, gene structure, gene expression, abiotic stress

## Abstract

Dehydration-responsive element-binding (DREB) transcription factors of the A2 subfamily play key roles in plant stress responses. In this study, we identified and characterized a new A2-type *DREB* gene, *ZmDREB2.9*, in the *Zea mays* cv. B73 genome and compared its expression profile with those of the known A2-type maize genes *ZmDREB2.1–2.8*. *ZmDREB2.9* was mapped to chromosome 8, contained 18 predicted hormone- and stress-responsive *cis*-elements in the promoter, and had two splice isoforms: short *ZmDREB2.9-S* preferentially expressed in the leaves, embryos, and endosperm and long *ZmDREB2.9-L* expressed mostly in the male flowers, stamens, and ovaries. Phylogenetically, ZmDREB2.9 was closer to *A. thaliana* DREB2A than the other ZmDREB2 factors. *ZmDREB2.9-S*, *ZmDREB2.2*, and *ZmDREB2.1/2A* were upregulated in response to cold, drought, and abscisic acid and may play redundant roles in maize stress resistance. *ZmDREB2.3*, *ZmDREB2.4*, and *ZmDREB2.6* were not expressed in seedlings and could be pseudogenes. *ZmDREB2.7* and *ZmDREB2.8* showed similar transcript accumulation in response to cold and abscisic acid and could be functionally redundant. Our results provide new data on *Z. mays* DREB2 factors, which can be used for further functional studies as well as in breeding programs to improve maize stress tolerance.

## 1. Introduction

Maize (*Zea mays* L.) is the third most extensively cultivated cereal crop worldwide. Its ability to cross-pollinate in combination with a large genome (2.3 Gb) [1,2] provides an exceptional level of genetic diversity, which is successfully exploited in breeding programs [3,4]. Maize has been the object of many studies on monocot evolution [5], epigenetics [6], transposition [7], heterosis [8], and chloroplast differentiation in C4 species [9]. However, the need for genetic improvement of maize agricultural and economic traits is constantly increasing, especially in view of expanding cultivation in different areas, and further studies on the mechanisms regulating plant resistance to various biotic and abiotic stresses are required.

External stress stimuli perceived by plant cells through cell wall receptors trigger intracellular signaling mainly associated with reactive oxygen species (ROS) production and regulation of free Ca^2+^ concentration. The two main stress-activated signaling cascades involving mitogen-activated protein kinases (MAPKs) and Ca^2+^-dependent protein kinases (CDPKs) [10] are coordinated by phytohormones such as abscisic acid (ABA), jasmonic acid (JA), salicylic acid (SA), and ethylene (ET) and controlled by transcription factors (TFs) [11,12]. The APETALA2/ET-responsive element binding factor (AP2/ERF) family includes ERFs and dehydration-responsive element-binding (DREB) proteins, which bind to ET-responsive elements via the GCC box (AGCCGCC) and to the *cis*-acting DRE/C-repeat site (TACCGACAT), respectively [13,14,15].

Based on the sequence similarity of their AP2 domains, DREB factors are divided into six types (A1–6) [14], all of which are implicated, albeit to a different extent, in the control of hormone and stress responses. Among *DREB* genes of the A3–6 types, *ABI4* (A3-type) has been shown to regulate ABA signaling in *Arabidopsis thaliana* and maize [16,17,18] and *A. thaliana TINY* (A4-type)—the response to drought, cold, and exogenous ET and JA [19]. The overexpression of *GhDBP3* (A4-type) or *GhDBP2* (A6-type) in *Gossipium hirsutum* [20,21] and *StDREB2* (A5-type) in *Solanum tuberosum* [22] is sufficient to increase plant resistance to abiotic stresses. At the same time, maize *ZmDREB4.1* (A4-type) is not induced by such stresses as drought, salt, cold, or wounding [23].

The largest and most studied types of DREB TFs are A1 (DREB1/C-repeat factor [CBF]) and A2 (DREB2), which are mostly involved in abiotic stress resistance [24,25,26,27]. DREB1/CBF and DREB2 factors have high homology in the DNA-binding domain characteristic for the AP2/ERF family and can bind the same DRE core sequence (A/GCCGAC). DREB1/CBFs are considered to be important for cold tolerance [28,29,30] and are conserved in plants regardless of their ability to acclimatize to cold [27,31]. The expression of *Arabidopsis DREB1/CBF* genes is sharply and strongly induced by low temperatures, whereas that of *DREB2A* genes is almost not affected by cold but upregulated in response to dehydration and salt stresses [14,30,32]. On the other hand, *Oryza sativa OsDREB1* gene is found to be induced not only by cold but also by high salinity and drought [32]. Functionally redundant *CBF1*–*CBF3* genes are quickly induced by low temperatures, and the corresponding proteins activate the expression of >40 cold-responsive genes regulated by *DREB1/CBF* [28,30,33,34,35,36]. In many plants, including maize, the overexpression of the DREB1/CBF regulon improves the resistance to frost [37,38,39].

DREB2 TFs regulate responses to a wider range of abiotic stresses, including drought, salt, heavy metals, cold, and heat [27,40,41,42]. Thus, *SbDREB2A* and *SbDREB2B* genes are activated in *Sorghum bicolor* treated with salt and cadmium [43], whereas the overexpression of *SbDREB2* in transgenic *Oryza sativa* increases plant resistance to water deficiency [44]. *Triticum aestivum TaDREB1* gene (A2-type) is found to be induced by cold, high salinity and drought [45]. *H. vulgare HvDRF1* (A2-type) is reported to accumulate after drought and high salinity stresses and to be involved in ABA-mediated gene regulation [46]. *DREB2A* and *DREB2B* are induced in *A. thaliana* after osmotic stress [30], and it has been shown that the two genes are major TFs regulating the expression of high salinity- and drought-induced genes, respectively [47,48].

Advances in whole-genome sequencing have facilitated the identification and characterization of AP2/ERF factors in plants, including maize [49,50]. A total of 65 members of the DREB family are classified in the genome of *Z. mays* cv. B73 based on a previous assembly (GCF_000005005.2), including 10 genes belonging to each of the canonical A1 and A2 types [49], and 61 DREB genes are predicted based on the current genome assembly (GCF_902167145.1) [50]. However, the information on structural and functional characteristics of maize A2-type genes is rather limited. A previous study has analyzed the expression pattern of *ZmDREB2.1–ZmDREB2.8* genes in different maize organs and revealed a significant association of the *ZmDREB2.7* promoter region with drought tolerance at the seedling stage [49]. *ZmDREB2A* is the most studied *DREB* gene in maize; it has two splice isoforms, of which only the functional one, *ZmDREB2A-S*, is significantly induced by abiotic stresses such as cold, heat, dehydration, and salt through upregulation of acetylated histones H3K9 and H4K5 associated with the *ZmDREB2A* promoter [51,52].

In this study, we identified and characterized a new A2-type *ZmDREB2.9* gene homologous to *ZmDREB2.1/2A* and compared its structural composition, promoter *cis*-regulatory elements, and expression pattern in maize organs with those of the known *ZmDREB2.1/2A–2.8* genes. The transcript accumulation of *ZmDREB2* genes in response to cold, salt, drought, and exogenous ABA were also analyzed. Our results provide new data on the DREB2 gene subfamily in *Z. mays*, which can be used in breeding programs to improve maize stress tolerance and acclimatization.

## 2. Results

### 2.1. Characterization of A2-Type DREB Genes in Z. mays B73 and Identification and Analysis of a New A2-Type ZmDREB2.9 Gene

The information on eight A2-type *ZmDREB* genes extracted from previous genome-wide identification reports [49,50] and the NCBI and Maize genome databases is summarized in Table 1. In this study, we identified a new A2-type *ZmDREB* gene based on BLAST-P alignment and characteristics of the A2-type DNA-binding domain [14]; this gene has not been previously described but is annotated in the NCBI as *ZmDREB2A* (Gene ID: 100286109; Table 1). It should be noted that another *Z. mays* gene has been widely studied under the same name, *ZmDREB2A*; however, in the NCBI it is annotated as *ZmDREB1c* (Gene ID: 732788), although *ZmDREB2A* is mentioned among the synonymous gene names (Table 1). To avoid confusion, we used the names *ZmDREB2.1/2A–ZmDREB2.8*, where *ZmDREB2.1/2A* corresponds to *ZmDREB1c* in [49], and designated the new gene as *ZmDREB2.9* (Table 1).

**Figure 1:**
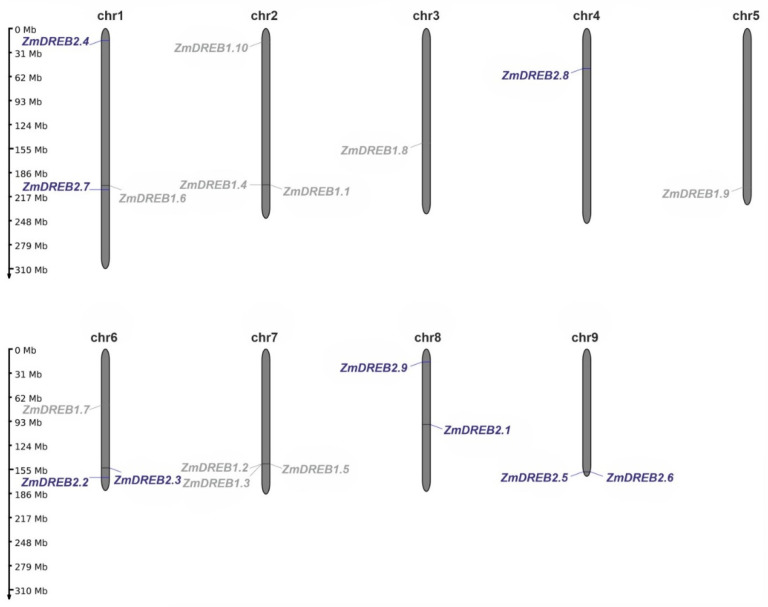
Chromosome location of A1-type *ZmDREB1/2A* (grey) and A2-type *ZmDREB2.1/2A–2.9* (blue) genes in the *Z. mays* genome. Chromosome lengths (indicated on the left) are based on the *Z. mays* cv. B73 genome (Zm-B73-REFERENCE-NAM-5.0); chr, chromosome.

The *ZmDREB2.1/2A–2.9* genes are evenly distributed over maize chromosomes 1, 4, 6, 8, and 9; similar to *ZmDREB2.1/2A*, the new *ZmDREB2.9* gene is located on chromosome 8 (Figure 1).

The main characteristics of the translated *ZmDREB2* gene products are presented in Table 1. All analyzed proteins had a full-length DNA-binding AP2 domain (smart00380), which contained V14 and E19 residues important for the DNA-binding specificity of DREB2A TFs [14].

**Figure 2:**
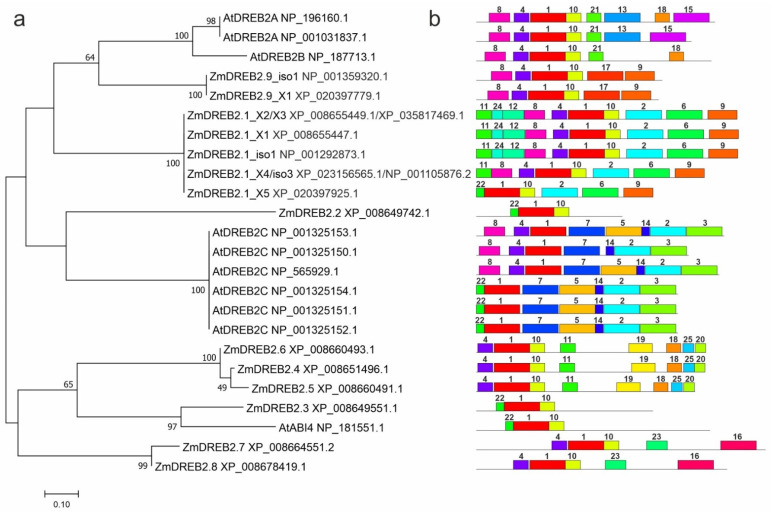
Evolutionary relationships of the ZmDREB2.1/2A–2.9 and *A. thaliana* AtDREB proteins (NCBI IDs are indicated). (**a**) The unrooted dendrogram was constructed using the Maximum Likelihood method according to the JTT matrix-based model (bootstrap test: 1000 replicates) in MEGA 7.0.26. (**b**) Distribution of conserved motifs in ZmDREB2.1/2A–2.9 and AtDREB proteins. Analysis was performed using MEME 5.4.1; the length of each box corresponds to that of the motif.

Phylogenetic analysis revealed three clades: DREB2A (ZmDREB2.1/2A and ZmDREB2.9), DREB2C (ZmDREB2.2), and ABI4 (ZmDREB2.3–2.8) (Figure 2a). Among the proteins of the DREB2A clade, the AP2 domain is a highly conserved region, while the N-terminus is the most polymorphic (Figure S1). ZmDREB2.9 was closer to DREB2A than ZmDREB2.1/2A. In the NCBI non-redundant protein sequence database, ZmDREB2.1/2A and ZmDREB2.9 are homologous to each other (identity 68%) and to the *A. thaliana* DREB2A protein (NP_001031837.1; identity 66% with both maize proteins).

A total of 25 conserved motifs were identified in the analyzed ZmDREB2 proteins and their *A. thaliana* homologs (Figure 2b). Motifs 1 and 10 constituted the AP2-domain. Proteins of the DREB2A clade shared motifs 8, 4, 1, and 10 (except for isoform X5 of ZmDREB2.1/2A, although motif 22 [consensus MKGKGGPENGI] was a part of motif 4 [RKAPAKGSKKGCMKGKGGPEN]). Motifs 21, 13, 15, and 18 were found in AtDREB2A/B proteins, motifs 17 and 9—in ZmDREB2.9, and motifs 11, 24, 12, 2, and 9—in ZmDREB2.1/2A. ZmDREB2.2 contained only motifs 22, 1, and 10 and could represent a truncated version or be a result of incorrect assembly. Motifs 19, 25, and 20 were unique for ZmDREB2.3–2.6 and motifs 23 and 16—for ZmDREB2.7 and ZmDREB2.8.

These results confirmed the phylogenetic relationship among ZmDREB2 and AtDREB2A proteins (Figure 2a), Thus, the motif profiles of ZmDREB2.9 (8-4-1-10-17-9) and AtDREB2A (8-4-1-10-21-13-[18]-15) showed the greatest similarity and differed from that of ZmDREB2.1/2A, which had additional N-terminal motifs (11-24-12), although containing the same C-terminal motif 9 as ZmDREB2.9 (Figure 2b).

### 2.2. ZmDREB2.1–2.9 Promoter Analysis

Considering the role of *ZmDREB2* genes in maize stress response [49,50,51], we searched for *cis*-acting elements in the 5′-UTR and promoter regions (1 kb upstream of the start codon). As a result, 6 hormone- and 9 stress-responsive elements and 9 other regulatory sites associated with developmental processes and TF binding were identified (Table 2). Among the former, the most common were ABA responsive elements (ABRE, detected in all genes except *ZmDREB2.3–2.5*) and the CGTCA motif (MeJA and osmotic stress responsiveness; detected in all genes except *ZmDREB2.3*); the most enriched for ABRE were *ZmDREB2.7* and *ZmDREB2.8* and for CGTCA—*ZmDREB2.7* and *ZmDREB2.9*. SA-responsive sites were found in *ZmDREB2.1/2A*, *ZmDREB2.4*, and *ZmDREB2.5*, auxin-responsive—in *ZmDREB2.1/2A* and *ZmDREB2.9*, and gibberellin-responsive—in *ZmDREB2.3* and *ZmDREB2.8*. ET-sensitive elements were not detected (Table 2).

Among the 9 stress-responsive elements, the most common were anaerobic responsive element (ARE) involved in the activation of anaerobic gene expression (the highest number in *ZmDREB2.4*) and stress response element (STRE) implicated in the regulation of heat and osmotic stress-related genes (the highest number in *ZmDREB2.1*). Drought-responsive elements (DRE1/DRE core) were found in the promoters of *ZmDREB2.2*, *ZmDREB2.7*, and *ZmDREB2.8* and low temperature-responsive elements (LTRs)—in those of *ZmDREB2.3* and *ZmDREB2.7*.

Comparison of the promoter regions in *ZmDREB2.1/2A* and *ZmDREB2.9* revealed 4 and 3 ABRE, 1 and 1 TGA element, 1 and 4 CGTCA motifs, 1 and 0 TCA elements, 2 and 1 AREs, 0 and 2 STREs, 0 and 1 W-box, 0 and 2 WRE3, and 0 and 2 GC motifs, respectively. Promoters of both genes had *cis*-elements related to meristem-specific activation (CCGTCC motif) and that of *ZmDREB2.1/2A* had a site associated with circadian control.

The *ZmDREB2.4* promoter contained 7 CAT-box elements, suggesting that this TF may be the most responsive to developmental processes.

### 2.3. ZmDREB2.9 Expression in Various Organs of Maize cv. B73

**Figure 3:**
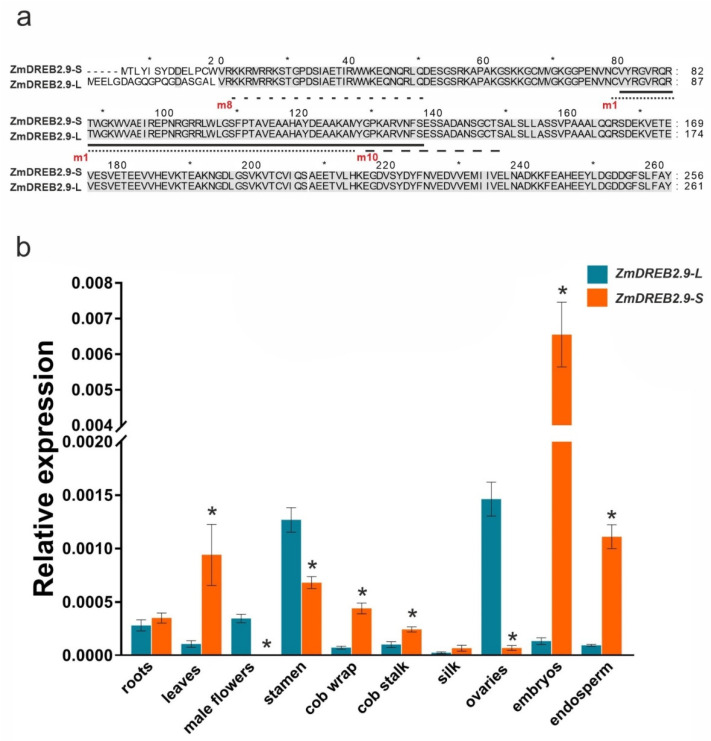
Comparison of *ZmDREB2.9* isoforms *ZmDREB2.9-L* and *ZmDREB2.9-S*. (**a**) Sequence alignment of *ZmDREB2.9-S* and *ZmDREB2.9-L*; 100% identical regions are highlighted gray, and the AP2 domain is underlined with a solid line. The sequences of motif 8 (m8; see Figure 2b), following the area of *ZmDREB2.9* alternative splicing, as well as motifs 1 (m1) and 10 (m10), comprising the AP2 domain, are shown by a dotted lines (**b**) Transcript levels of *ZmDREB2.9-L* and *ZmDREB2.9-S* in the indicated *Z. mays* cv. B73 tissues. The data were normalized to the mRNA expression of the *ZmUBC* gene (GRMZM2G419891) and presented as the mean ± SD (*n* = 3); * *p* < 0.01.

To elucidate the role of the identified *ZmDREB2.9* gene in maize development, we analyzed the transcript levels of two *ZmDREB2.9* isoforms: long form iso1 (*ZmDREB2.9-L*; NM_001372391.2) and short form X1 (*ZmDREB2.9-S*; XM_020542190.3) in vegetative and reproductive tissues by quantitative real-time (qRT) PCR (Figure 3a). The results showed that both isoforms were expressed in all analyzed organs (Figure 3b). *ZmDREB2.9-S* was most strongly expressed in embryos, where *ZmDREB2.9-L* transcripts were present in very low numbers. The expression of *ZmDREB2.9-S* was also significantly higher than that of *ZmDREB2.9-L* in the endosperm, cob wraps and stalks, and leaves and lower in the stamens, ovaries, and male flowers.

### 2.4. ZmDREB2.1–2.9 Expression in Maize Seedlings in Response to Stresses

**Figure 4:**
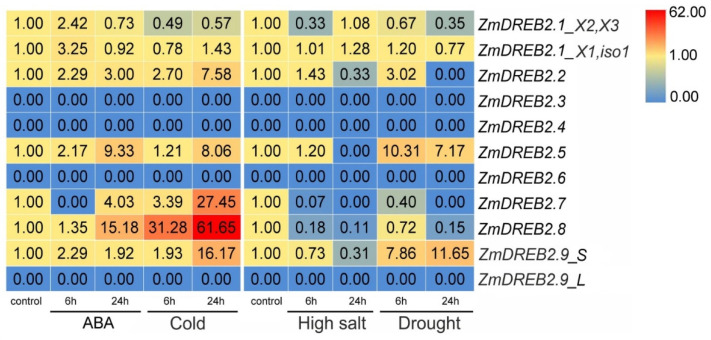
Heatmap of *ZmDREB2.1/2A–2.9* time-dependent expression in *Z. mays* cv. B73 seedlings subjected to cold, high salt, and drought stresses and ABA treatment. The data were normalized to *ZmUBC* expression. The color gradient indicates expression changes from low (blue) to high (red).

Considering the different profiles of stress-responsive *cis*-regulatory elements in *ZmDREB2.1/2A–2.9* promoters (Table 2), we analyzed the expression of these genes in the leaves of maize seedlings exposed to cold, salt, drought, and exogenous ABA at 6 and 24 h after treatment (Figure 4). After 6 h, the experimental plants did not differ outwardly from the control, while after 24 h a slight wilting of the leaves was observed in the case of cold, salinity and drought; we did not notice any difference with the control when treated with ABA (Figure S2). Leaves analyzed for relative water content (RWC) at 6/24 h points were characterized by a decrease in water content of ~4/5% (ABA), ~5/13% (cold), ~5/18% (salinity) and ~11/21% (drought) (Figure S3).

The results revealed that *ZmDREB2.3*, *ZmDREB2.4*, and *ZmDREB2.6* were not transcribed in the young leaves either under normal or stress conditions (Figure 4).

For *ZmDREB2.1/2A*, we evaluated the transcript levels of four isoforms, X1/iso1 and X2/X3 (Table 1); as X4 and iso3 corresponded to *ZmDREB2A-S*, which was analyzed previously for stress response [51], they were not analyzed. The identity of the shortest isoform X5 to the corresponding section of the *ZmDREB2.1/2A* isoforms did not allow the design of the primers to test its expression. The expression of X2/X3 was downregulated by cold, high salt, and drought, and slightly upregulated by ABA at 6 h. Iso1/X1 expression was also upregulated at 6 h after ABA treatment but not affected by stresses (Figure 4).

*ZmDREB2.2* and *ZmDREB2.5* were upregulated by ABA and cold and showed bell-shaped expression changes (increase at 6 h and decrease at 24 h) after exposure to high salt and drought (Figure 4).

*ZmDREB2.7* was repressed by salt and drought but strongly activated by cold; it was downregulated by ABA at 6 h and upregulated at 24 h (Figure 4).

*ZmDREB2.8* transcript accumulation was significantly induced by ABA and especially by cold stress and downregulated by salt and drought (Figure 4).

*ZmDREB2.9-L* transcripts were not detected in the seedlings under normal or stress conditions. However, *ZmDREB2.9-S* was upregulated by cold, drought, and, to a lesser extent, by ABA and downregulated by high salt (Figure 4).

## 3. Discussion

The development and productivity of plants are affected by various abiotic stresses, which activate plant molecular mechanisms providing adaption to adverse conditions. Drought, high salinity, and extreme temperatures limit the geographical distribution of plants as they cause dehydration and, ultimately, cell death [53]. Chilling (0–15 °C) increases membrane rigidity, destabilizes protein complexes, and disrupts photosynthesis [54], and freezing (<0 °C) results in ice formation in the apoplast [55] and destruction of cellular membranes [28]. Drought leads to dehydration resulting in osmotic and oxidative stresses and cell death [56], whereas high salinity reduces water uptake, causing toxic effects, nutritional imbalance, and acceleration of ROS production [57].

DREB TFs are key regulators of plant responses to stressful conditions [27,53], playing an important role in the protection and acclimation of various plant species, including maize [49,51,52], which suggests their potential utility in crop breeding programs. In the present study, we identified and characterized a new maize gene, *ZmDREB2.9*, belonging to the A2-type DREB subfamily and performed comparative profiling of *DREB2* maize genes in terms of tissue expression patterns and stress-dependent regulation.

Although *ZmDREB2.9* has been annotated in the NCBI as *ZmDREB2A*, it has not yet been studied, probably because of confusing terminology, since the *ZmDREB2.1/2A* gene (annotated as *ZmDREB1c* in the NCBI) is already known in the literature. Our phylogenetic analysis indicates that the products of the new *ZmDREB2.9* and the known *ZmDREB2.1/2A* genes are structural homologs and belong to the AtDREB2A/B clade (Figure 2a), suggesting similar functions in the regulation of abiotic stress responses [27,51,58,59,60]. Furthermore, *ZmDREB2.9* and *ZmDREB2.1/2A* are located on the same chromosome 8 (Figure 1) and could possibly be a result of segmented gene duplication, implying their functional redundancy.

Unlike the intronless *ZmDREB2.3–2.8* genes, *ZmDREB2.9* and *ZmDREB2.1/2A* contain 1 and 1–3 (depending on the splicing scheme) introns, respectively. Considering that intronless genes have evolved from their intron-containing counterparts in order to accelerate plant stress response [61], it can be assumed that *ZmDREB2.9* and *ZmDREB2.1/2A* have a more ancient origin than *ZmDREB2.3–2.8*. Interestingly, *ZmDREB2.3–2.8* are homologous to *ABI4* (Figure 2a), which is involved in ABA signaling [16,17,18] and is considered an A3-type DREB factor, which questions the assignment of ZmDREB2.3–2.8 to the A2 subfamily [49].

The *ZmDREB2.9* gene produces two transcript variants, longer *ZmDREB2.9-L* and shorter *ZmDREB2.9-S* (Figure 3a), similar to *ZmDREB2.1/2A* and *AtDREB2A*, which also have long and short transcript isoforms (Table 1). Among the *ZmDREB2.1/2A* splicing isoforms, only a shorter one, *ZmDREB2.1/2A-S* (AB218832, homologous to X4 and iso 3; Table 1), is considered to be functional and is significantly induced by temperature, drought, and osmotic stresses [27,51,58]. Consistent with these data, we observed stronger expression of the shorter *ZmDREB2.9-S* isoform compared to the longer one, *ZmDREB2.9-L*, although both transcripts were detected in maize adult tissues (Figure 3b). Furthermore, only *ZmDREB2.9-S* transcript accumulation was affected by cold, high salt, drought, and external ABA (Figure 4). These results imply functional similarity between *ZmDREB2.1/2A-S* and *ZmDREB2.9-S*.

It has been suggested that the transcriptional activity of *AtDREB2A* in *A. thaliana* depends on the stability of the protein product; as the short form AtDREB2A-CA lacking a 30 aa region (located between the AP2 domain and C-terminus) is stable, it can positively affect the level of the corresponding transcript [59,60]. In view of this, it can be hypothesized that the difference in the expression between shorter and longer isoforms of *ZmDREB2.1/2A* and *ZmDREB2.9* could be attributed to the length of the N-terminus in the corresponding proteins (Figure 2b and Figure 3a), which may affect protein stability and, ultimately, isoform transcript accumulation. MEME motif profiling revealed that the N-terminus of the shorter ZmDREB2.1/2A isoforms differed from that of the longer ones (which are not functional [51]) by the absence of motifs 24 and 12; however, the motif composition of ZmDREB2.9-L was identical to that of ZmDREB2.9-S (Figure 2b). Therefore, in contrast to ZmDREB2.1/2A-L, ZmDREB2.9-L may be functional, which is consistent with its expression pattern in maize organs, including male flowers, stamens, and ovaries, where its mRNA levels even exceeded those of the shorter form (Figure 3).

The increased expression of *ZmDREB2.9-S* in embryos and endosperm (Figure 3b) indicates its possible role in grain development and maturation, which may be related to ABA accumulation. ABA is known to promote cell division in the seed endosperm and increase grain capacity, filling rate, and yield [62,63]. In this study, we found that the expression of *ZmDREB2.9-S* was upregulated in response to ABA (Figure 4), which can be attributed to the presence of three ABRE elements in the promoter (Table 2).

We observed differential stress responses of homologous *ZmDREB2.9* and *ZmDREB2.1/2A* genes, which could be associated with the differences in *cis*-regulatory motif profiles of their promoters. Although both genes had a similar set of promoter hormone-sensitive elements, *ZmDREB2.9* had a higher number of stress-sensitive motifs, whose pattern was more similar to that of *ZmDREB2.2* (Table 2). The *ZmDREB2.2* gene, more distantly related to the *DREBA/B* group (Figure 2a), was activated in response to ABA, cold, and drought (Figure 4), suggesting its role in maize stress resistance, which could be redundant to those of *ZmDREB2.9* and *ZmDREB2.1/2A*.

Stress-sensitive elements were also found in the promoters of the *ABI4* clade genes, *ZmDREB2.3*, *ZmDREB2.4*, and *ZmDREB2.6* (Table 2). However, these genes were not transcribed in maize seedlings either under normal or stress conditions (Figure 4), although *ZmDREB2.3* and *ZmDREB2.4* have been previously shown to be expressed, albeit at low levels, in seedlings under normal conditions [49]. These data suggest that *ZmDREB2.3*, *ZmDREB2.4*, and *ZmDREB2.6* may be pseudogenes or have a yet-unknown function. It should be noted that the *ZmDREB2.4* gene is currently not annotated in the NCBI database (Table 1), and it is unclear which gene is expressed, and whether the *ZmDREB2.3* gene is transcribed in fact [49].

A decrease in *ZmDREB2.7* expression in response to drought (Figure 4) is consistent with the association between dehydration resistance at the seedling stage and the *ZmDREB2.7* promoter structure [49]. At the same time, *ZmDREB2.7* transcript level was significantly activated by ABA and cold and inhibited by high salinity (Figure 4), which corresponds with the assignment of *ZmDREB2.7* to the *ABI4* group (Figure 2a) and may indicate its involvement in the ABA signaling pathway and maize resistance to abiotic stresses. The function of the *ZmDREB2.7* gene may be redundant to that of *ZmDREB2.8*, which was identified as the closest *ZmDREB2.7* homolog (Figure 2a) and behaved similarly under stresses (Figure 4). It is possible that the *ZmDREB2.7* and *ZmDREB2.8* genes may have emerged through gene duplication. *ZmDREB2.7* is the only gene whose promoter has a zein metabolism regulation element (O2 site) (Table 2), and it can be hypothesized that *ZmDREB2.7* could have acquired (or *ZmDREB2.8* lost) the O2 site through neofunctionalization; thus, *ZmDREB2.7* may be involved in the regulation of zein metabolism in maize grain.

Our results provide new data about the *Z. mays DREB* A2-type genes, which can be useful for breeding programs aimed on increasing the resistance of maize crop to various abiotic stresses.

## 4. Materials and Methods

### 4.1. In Silico Identification and Structural Characterization of ZmDREB2 Genes

The search for *ZmDREB2* genes was performed based on the *Z. mays* cv. B73 whole-genome assembly (NCBI *Zea mays* Annotation Release 103; GCF_902167145.1) and previous publications [49,50].

Multiple sequence alignment, structural analyses of the *ZmDREB2* genes and encoded proteins, and construction of a phylogenetic dendrogram (Maximum Likelihood method) were conducted using MEGA 7.0.26 [64]; confidence for tree topologies was estimated by bootstrap values of 1000 replicates.

Putative ZmDREB proteins were characterized by molecular weight, pI (ExPASy ProtParam; https://web.expasy.org/protparam/; accessed on 30 August 2022), conserved domains, sites, and motifs (NCBI-CDD, https://www.ncbi.nlm.nih.gov/cdd; accessed on 30 August 2022; and MEME 5.4.1, http://meme-suite.org/tools/meme; accessed on 30 August 2022). The chromosomal localization map was drawn using MG2C v. 2.1 (http://mg2c.iask.in/mg2c_v2.1/; accessed on 30 August 2022).

### 4.2. RNA Extraction and qRT-PCR

Total RNA was extracted from individual roots, leaves, male flowers, stamens, cob wraps, cob stalks, silk, ovaries, grain embryos, and grain endosperm (0.1 g of each tissue) using the RNeasy Plant Mini Kit (QIAGEN, Hilden, Germany), purified from genomic DNA (RNase free DNase set; QIAGEN), qualified by gel electrophoresis, and used for first-strand cDNA synthesis (GoScript Reverse Transcription System; Promega, Madison, USA) with an oligo-dT primer. RNA and cDNA concentrations were quantified by fluorimetry (Qubit^®^ Fluorometer, Thermo Fisher Scientific, Waltham, MA, USA), and qRT-PCR was performed in a CFX96 Real-Time PCR Detection System (Bio-Rad Laboratories, Hercules, CA, USA) with 3.0 ng cDNA, SYBR Green RT-PCR mixture (Syntol, Moscow, Russia), and specific primers (Table S1). The following cycling conditions were used: initial denaturation at 95 °C for 5 min, 40 cycles of denaturation at 95 °C for 15 s, and annealing/extension at 60 °C for 40 s.

*ZmDREB2* gene expression was normalized using the *ZmUBC* gene (GRMZM2G419891) as reference [49], and the qRT-PCR results were statistically analyzed with Graph Pad Prism version 8 (GraphPad Software Inc., San Diego, CA, USA; https://www.graphpad.com/scientific-software/prism/, accessed on 27 July 2022). The data were expressed as the mean ± standard deviation (SD) based on three technical replicates of three biological replicates for each combination of cDNA and primer pairs. The unequal variance (Welch’s) *t*-test was applied to assess differences in gene expression; *p* < 0.01 was considered to indicate statistical significance.

### 4.3. Promoter and 5′-UTR Analysis

The search for specific *cis*-elements in the promoters and 5′-UTRs (1.0 kb regions upstream of the initiation codon) was performed using the PlantCARE database, which provides evaluation of *cis*-regulatory elements, enhancers, and repressors; (http://bioinformatics.psb.ugent.be/webtools/plantcare/html/; accessed on 25 August 2022).

### 4.4. Plant Material and Stress Assays

*Z. mays* cv. B73 grains were germinated in pots with soil, and plant were grown in a greenhouse (16 h light/8 h dark; 23 °C) for two weeks (stress analysis) and two and a half months (to the harvest; for the collection of tissues of various organs). Total RNA was isolated from adult plant organs (roots, leaves, male flowers, stamens, cob wrap, cob stalk, silk, ovaries, and grain embryos and endosperm) and used to synthesize cDNA for gene expression analysis by qRT-PCR.

Maize seedlings at the stage of 3–4 leaves were used to analyze stress response. Cold stress was performed at +4 °C in a climatic chamber. ABA treatment was done by spraying seedlings with 100 µM ABA. To analyze the effect of drought and high salt, seedlings with roots were cleaned from soil with distilled water and transferred to liquid Murashige and Skoog medium supplemented with 10% polyethylene glycol (PEG-6000) or 250 mM NaCl, respectively.

Leaves were harvested 6 and 24 h after each treatment and frozen in liquid nitrogen until further analyses. Untreated plants were used as control. The experiments were performed in two biological and three technical replicates.

Plant leaves at points 6 and 24 h post-stress were analyzed for RWC according to [65]. To do this, the leaf (petiole to the bottom) was placed in a pre-weighed airtight vial and weighed to obtain the weight of the leaf sample (W). Then, the sample was moistened for 3–4 h at room light and temperature: deionized water was poured into the vial to a level of 2 cm, the vial was closed with a lid, and the leaf received moisture through the petiole. After hydration, the samples were dried from surface moisture using filter paper and weighed to obtain the total turgid mass (TW). The samples were then dried in an oven at 80 °C for 24 h and after cooling were weighed to determine the dry weight (DW). Calculation: RWC (%) = [(W − DW)/(TW − DW)] × 100.

## 5. Conclusions

In the *Z. mays* cv. B73 genome, we identified and characterized a new A2-type *ZmDREB2.9* gene, which showed homology to the *ZmDREB2.1/2A* gene, and compared its expression profile with those of the known A2-type maize genes *ZmDREB2.1/2A–2.8*. The two *ZmDREB2.9* splice isoforms had distinct expression patterns in maize organs, indicating preferential involvement of the shorter transcript *ZmDREB2.9-S* in the development of the leaves, embryos, and endosperm and that of the longer transcript *ZmDREB2.9-L* in the development of the male flowers, stamens, and ovaries. Analysis of protein sequence homology, transcriptional response to stresses, and profiles of promoter hormone- and stress-responsible *cis*-acting elements points on the functional redundancy of *ZmDREB2.9-S*, *ZmDREB2.1/2A*, and *ZmDREB2.2* as A2-type *DREB* genes. The absence of *ZmDREB2.3*, *ZmDREB2.4*, and *ZmDREB2.6* transcripts in maize seedlings both under normal and stress conditions suggests that they are either pseudogenes or have an unknown function. The *ZmDREB2.7* gene may regulate zein metabolism in maize grain and, together with *ZmDREB2.8*, play a redundant role in ABA signaling and plant resistance to abiotic stresses. Our results provide new data on the A2-type DREB TFs in *Z. mays*, which can be used for further functional characterization of the *ZmDREB2.1/2A–2.9* genes and could contribute to the development of breeding programs to improve maize stress tolerance and acclimatization.

## Figures and Tables

**Table 1:** Characteristics of the A2-type *DREB* genes in the *Z. mays* B73 genome.

Gene Name *	Gene/Locus ID	Genomic Localization (NCBI)	Gene, bp	CDS, bp	Protein, aa	MW, kDa	pI	AP2 Domain Localization, aa	Annotation in Zm-B73-REFERENCE-NAM-5.0
*ZmDREB2.1*/no	Gene ID: 732788 LOC732788/Zm00001d010048/GRMZM2G006745	chr8:96775353–96778297 [NC_050103.1 (96775198..96778545)]	2945	1107 (X1)	368 XP_008655447.1	39.65	5.08	134–190	DRE-binding protein 1c, also known as DBP1a; DBP1b; DBP1c; DREB2; DREB2A; TIDP2952; ZmDREB2A; GRMZM2G006745
*ZmDREB2.1*/*ZmERF135*				1104 (X2)	367 XP_008655449.1	39.58	5.08	133–189	
*ZmDREB2.1*/no				1101 (X3)	366 XP_035817469.1	39.51	5.02	132–188	
*ZmDREB2.1*/*ZmERF134*				963 (X4)	320 XP_023156565.1	34.7	4.94	86–142	
*ZmDREB2.1*/*ZmERF136*				747 (X5)	248 XP_020397925.1	26.55	4.3	14–70	
*ZmDREB2.1*/no				1104 (iso1)	367 NP_001292873.1	39.58	5.02	133–189	
*ZmDREB2.1*/no				960 (iso3)	319 NP_001105876.2	34.62	4.94	85–141	
*ZmDREB2.2*/no	Gene ID: 103630470 LOC103630470	chr6:164425309–164427359 [NC_050101.1 (164425038..164427713)]	2051	618	205 XP_008649742.1	22.01	6.35	61–118	Dehydration-responsive element-binding protein 2D
*ZmDREB2.3*/*ZmERF104*	Gene ID: 100384333 LOC100384333/Zm00001d038001/GRMZM2G093595	chr6:151835642–151836388 [NC_050101.1 (151835246..151836529, complement)]	747	747	248 XP_008649551.1	26.8	7.99	41–103	Ethylene-responsive transcription factor ABI4
*ZmDREB2.4*/no	Gene ID: 103631782 LOC103631782/Zm00001eb005650/GRMZM2G419901	chr1:15900859–15901824 [NC_024459.2 (16005938..16006903) B73 RefGen_v4 (GCF_000005005.2)]	966	966	321 XP_008651496.1	34.08	5.99	28–89	Dehydration-responsive element-binding protein 2E Gene ID: 103631782, This record represents a gene not currently annotated in the NCBI.
*ZmDREB2.5*/*ZmERF154*	Gene ID: 103639528 LOC103639528/Zm00001d048296/GRMZM2G376255	chr9:157327869–157328792 [NC_050104.1 (157327689..157328926, complement)]	924	924	307 XP_008660491.1	32.72	5.65	28–90	Dehydration-responsive element-binding protein 2E
*ZmDREB2.6*/*ZmERF155*	Gene ID: 103639531 LOC103639531/Zm00001d048297/GRMZM2G399098	chr9:157365710–157366678 [NC_050104.1 (157365710..157366678, complement)]	969	969	322 XP_008660493.1	34.3	6.89	28–87	Dehydration-responsive element-binding protein 2E
*ZmDREB2.7*/*ZmERF18*	Gene ID: 103643169 LOC103643169/Zm00001d031861/GRMZM2G028386	chr1:206336830–206338050 [NC_050096.1 (206336803..206338449)]	1221	1221	406 XP_008664551.2	43.65	6.88	132–188	Ethylene-responsive transcription factor ABI4 (A3 subgroup)
*ZmDREB2.8*/*ZmERF57*	Gene ID: 103653247 LOC103653247/Zm00001d049889/GRMZM2G156737	chr4:51083167–51084225 [NC_050099.1 (51082881..51084560)]	1059	1059	352 XP_008678419.1	37.88	7.7	78–139	Dehydration-responsive element-binding protein 2C
*ZmDREB2.9*/no	Gene ID: 100286109 LOC100286109/ Zm00001d008665	chr8:16438393–16439714 [NC_050103.1 (16437969..16440069)]	1322	786 (iso1—L)	261 (L) NP_001359320.1	28.7	5.18	81–142	Dehydration-responsive element-binding protein 2A
				771 (X1—S)	256 (S) XP_020397779.1	28.65	5.26	76–137	

Note: * According to [49,50].

**Table 2:** Hormone- and stress-responsive *cis*-elements in the *ZmDREB2.1/2A–2.9* regulatory regions (~1000 bp). The color scheme (pale to dark) corresponds to the number of *cis*-elements (low to high).

Function	Element	Annotation	*ZmDREB2.1* */2A*	*ZmDREB2.2*	*ZmDREB2.3*	*ZmDREB2.4*	*ZmDREB2.5*	*ZmDREB2.6*	*ZmDREB2.7*	*ZmDREB2.8*	*ZmDREB2.9*
Hormone response	ABRE	*cis*-acting elements involved in ABA responsiveness	4	3				1	7	8	3
	CARE										
	AuxRR-core	*cis*-acting regulatory elements involved in auxin responsiveness									
	TGA element		1								1
	CGTCA motif	*cis*-acting regulatory element involved in MeJA-responsiveness	1	1		2	1	2	4	1	4
	SARE	*cis*-acting elements involved in SA responsiveness									
	TCA-element		1			1	1				
	P-box	gibberellin-responsive elements									
	TATC-box									1	
	GARE motif				1					1	
	ERE	ET-responsive element									
Stress response	ARE	*cis*-acting regulatory element essential for the anaerobic induction	2	2		4	2	1			1
	DRE1/DRE core	*cis*-acting regulatory element involved in drought response		1					1	1	
	LTR	*cis*-acting element involved in low-temperature responsiveness			1				1		
	STRE	*cis*-acting element involved in heat, osmotic stress, low pH, nutrient starvation stress response		7	2	2			3	1	2
	TC-rich repeats	*cis*-acting element involved in defense and stress responsiveness				2	1	1			
	W-box	WRKY-binding site involved in abiotic stress and defense response		1	2			1			1
	Wun motif	*cis*-acting elements involved in wounding and pathogen response			2						
	WRE3			1	1	1				1	2
	Box S										
	GC motif	enhancer-like element involved in anoxic specific inducibility			1	2		1	1		2
Developmental processes	O2-site	*cis*-acting element involved in zein metabolism regulation								1	
	CCGTCC motif	*cis*-acting element involved in meristem specific activation	1	1			1		3		2
	circadian	*cis*-acting element involved in circadian control	1								
	CAT-box	*cis*-acting regulatory element related to meristem expression	1	2		7		2			
	RY-element	*cis*-acting regulatory element involved in seed-specific regulation			2		2	2	1		
	MSA-like	*cis*-acting element involved in cell cycle regulation				1		1			
Other *cis*-elements	CCAAT-box/MYB/MRE	MYB-binding site	4	4	5	4	1	1	1	2	1
	MYC	MYC-binding site	1	2		2		2	2	2	3
	5′-UTR Py-rich stretch	*cis*-acting element conferring high transcript levels	1								

## Data Availability

*ZmDREB2.9* sequences are available in the NCBI database (see Table 1).

## Supplementary Materials

The following supporting information can be downloaded at: https://www.mdpi.com/article/10.3390/plants11223060/s1, Figure S1. Alignment of ZmDREB2.1/2A, ZmDREB2.9 and AtDREB2A isoforms (indicated according to Table 1). Figure S2. Photographs of maize seedlings 24 h after stresses (cold, salinity [NaCl], or drought [PEG]) compared to the untreated control. Seedlings after treatment with ABA are not shown, since outwardly, they did not differ from the control. Figure S3. Relative water content in the leaf of corn seedlings 6 and 24 h after stresses (ABA treatment, cold, salinity [NaCl], drought [PEG]) compared to the untreated control. Table S1: List of primers for *ZmDREB2.1/2A–2.9* gene expression analysis.

## References

[1] Matsuoka Y., Vigouroux Y., Goodman M., Sanchez G., Buckler E., Doebley J. (2002). A single domestication for maize shown by multilocus microsatellite genotyping. Proc. Natl. Acad. Sci. USA.

[2] Schnable P., Ware D., Fulton R., Stein J., Wei F., Pasternak S., Liang C., Zhang J., Fulton L., Graves T. (2009). The B73 maize genome: Complexity, diversity, and dynamics. Science.

[3] Diaw Y., Tollon-Cordet C., Charcosset A., Nicolas S.D., Madur D., Ronfort J., David J., Gouesnard B. (2021). Genetic diversity of maize landraces from the South-West of France. PLoS ONE.

[4] Josia C., Mashingaidze K., Amelework A.B., Kondwakwenda A., Musvosvi C., Sibiya J. (2021). SNP-based assessment of genetic purity and diversity in maize hybrid breeding. PLoS ONE.

[5] Doebley J. (2004). The genetics of maize evolution. Annu. Rev. Genet..

[6] Marand A.P., Chen Z., Gallavotti A., Schmitz R.J. (2021). A *cis*-regulatory atlas in maize at single-cell resolution. Cell.

[7] Stitzer M.C., Anderson S.N., Springer N.M., Ross-Ibarra J. (2021). The genomic ecosystem of transposable elements in maize. PLoS Genet..

[8] Xiao Y., Jiang S., Cheng Q., Wang X., Yan J., Zhang R., Qiao F., Ma C., Luo J., Li W. (2021). The genetic mechanism of heterosis utilization in maize improvement. Genome Biol..

[9] Majeran W., van Wijk K.J. (2009). Cell-type-specific differentiation of chloroplasts in C4 plants. Trends Plant Sci..

[10] Wurzinger B., Mair A., Pfister B., Teige M. (2011). Cross-talk of calcium-dependent protein kinase and MAP kinase signaling. Plant Signal. Behav..

[11] Erpen L., Devi H.S., Grosser J.W., Dutt M. (2018). Potential use of the DREB/ERF, MYB, NAC and WRKY transcription factors to improve abiotic and biotic stress in transgenic plants. Plant Cell Tissue Organ Cult..

[12] Wang J., Song L., Gong X., Xu J., Li M. (2020). Functions of jasmonic acid in plant regulation and response to abiotic stress. Int. J. Mol. Sci..

[13] Baillo E.H., Kimotho R.N., Zhang Z., Xu P. (2019). Transcription factors associated with abiotic and biotic stress tolerance and their potential for crops improvement. Genes.

[14] Sakuma Y., Liu Q., Dubouzet J.G., Abe H., Yamaguchi-Shinozaki K., Shinozaki K. (2002). DNA-binding specificity of the ERF/AP2 domain of *Arabidopsis* DREBs, transcription factors involved in dehydration- and cold-inducible gene expression. Biochem. Biophys. Res. Commun..

[15] Yamaguchi-Shinozaki K., Shinozaki K. (1994). A novel *cis*-acting element in an *Arabidopsis* gene is involved in responsiveness to drought, low-temperature, or high-salt stress. Plant Cell.

[16] Finkelstein R.R., Wang M.L., Lynch T.J., Rao S., Goodman H.M. (1998). The *Arabidopsis* abscisic acid response locus *ABI4* encodes an APETALA 2 domain protein. Plant Cell.

[17] Niu X., Helentjaris T., Bate N.J. (2002). Maize ABI4 binds coupling element1 in abscisic acid and sugar response genes. Plant Cell.

[18] Söderman E.M., Brocard I.M., Lynch T.J., Finkelstein R.R. (2000). Regulation and function of the *Arabidopsis ABA-insensitive 4* gene in seed and abscisic acid response signaling networks. Plant Physiol..

[19] Wilson K., Long D., Swinburne J., Coupland G. (1996). A Dissociation insertion causes a semidominant mutation that increases expression of *TINY*, an *Arabidopsis* gene related to *APETALA2*. Plant Cell.

[20] Huang B., Liu J.Y. (2006). Cloning and functional analysis of the novel gene *GhDBP3* encoding a DRE-binding transcription factor from *Gossypium hirsutum*. Biochim. Biophys. Acta.

[21] Huang B., Jin L., Liu J.Y. (2008). Identification and characterization of the novel gene *GhDBP2* encoding a DRE-binding protein from cotton (*Gossypium hirsutum*). J. Plant Physiol..

[22] Bouaziz D., Pirrello J., Ben Amor H., Hammami A., Charfeddine M., Dhieb A., Bouzayen M., Gargouri-Bouzid R. (2012). Ectopic expression of dehydration responsive element binding proteins (StDREB2) confers higher tolerance to salt stress in potato. Plant Physiol. Biochem..

[23] Li S., Zhao Q., Zhu D., Yu J. (2018). A DREB-Like transcription factor from maize (*Zea mays*), ZmDREB4.1, plays a negative role in plant growth and development. Front. Plant Sci..

[24] Hrmova M., Hussain S.S. (2021). Plant transcription factors involved in drought and associated stresses. Int. J. Mol. Sci..

[25] Lakhwani D., Pandey A., Dhar Y.V., Bag S.K., Trivedi P.K., Asif M.H. (2016). Genome-wide analysis of the AP2/ERF family in Musa species reveals divergence and neofunctionalisation during evolution. Sci. Rep..

[26] Liu Z., Yuan G., Liu S., Jia J., Cheng L., Qi D., Shen S., Peng X., Liu G. (2017). Identified of a novel *cis*-element regulating the alternative splicing of *LcDREB2*. Sci. Rep..

[27] Mizoi J., Shinozaki K., Yamaguchi-Shinozaki K. (2012). AP2/ERF family transcription factors in plant abiotic stress responses. Biochim. Biophys. Acta.

[28] Shi Y., Ding Y., Yang S. (2018). Molecular Regulation of CBF Signaling in Cold Acclimation. Trends Plant Sci..

[29] Jaglo-Ottosen K.R., Gilmour S.J., Zarka D.G., Schabenberger O., Thomashow M.F. (1998). *Arabidopsis CBF1* overexpression induces *COR* genes and enhances freezing tolerance. Science.

[30] Liu Q., Kasuga M., Sakuma Y., Abe H., Miura S., Yamaguchi-Shinozaki K., Shinozaki K. (1998). Two transcription factors, DREB1 and DREB2, with an EREBP/AP2 DNA binding domain separate two cellular signal transduction pathways in drought- and low-temperature-responsive gene expression, respectively, in *Arabidopsis*. Plant Cell.

[31] Agarwal P.K., Agarwal P., Reddy M.K., Sopory S.K. (2006). Role of DREB transcription factors in abiotic and biotic stress tolerance in plants. Plant Cell Rep..

[32] Dubouzet J.G., Sakuma Y., Ito Y., Kasuga M., Dubouzet E.G., Miura S., Seki M., Shinozaki K., Yamaguchi-Shinozaki K. (2003). *OsDREB* genes in rice, *Oryza sativa* L., encode transcription activators that function in drought-, high-salt- and cold-responsive gene expression. Plant J..

[33] Fowler S., Thomashow M.F. (2002). *Arabidopsis* transcriptome profiling indicates that multiple regulatory pathways are activated during cold acclimation in addition to the CBF cold response pathway. Plant Cell.

[34] Maruyama K., Sakuma Y., Kasuga M., Ito Y., Seki M., Goda H., Shimada Y., Yoshida S., Shinozaki K., Yamaguchi-Shinozaki K. (2004). Identification of cold-inducible downstream genes of the *Arabidopsis* DREB1A/CBF3 transcriptional factor using two microarray systems. Plant J..

[35] Park S., Lee C.M., Doherty C.J., Gilmour S.J., Kim Y., Thomashow M.F. (2015). Regulation of the *Arabidopsis* CBF regulon by a complex low-temperature regulatory network. Plant J..

[36] Jia Y., Ding Y., Shi Y., Zhang X., Gong Z., Yang S. (2016). The cbfs triple mutants reveal the essential functions of CBFs in cold acclimation and allow the definition of CBF regulons in *Arabidopsis*. New Phytol..

[37] Qin F., Sakuma Y., Li J., Liu Q., Li Y.Q., Shinozaki K., Yamaguchi-Shinozaki K. (2004). Cloning and functional analysis of a novel DREB1/CBF transcription factor involved in cold-responsive gene expression in *Zea mays* L.. Plant Cell Physiol..

[38] Ito Y., Katsura K., Maruyama K., Taji T., Kobayashi M., Seki M., Shinozaki K., Yamaguchi-Shinozaki K. (2006). Functional analysis of rice DREB1/CBF-type transcription factors involved in cold-responsive gene expression in transgenic rice. Plant Cell Physiol..

[39] Zhang X., Fowler S.G., Cheng H., Lou Y., Rhee S.Y., Stockinger E.J., Thomashow M.F. (2004). Freezing-sensitive tomato has a functional CBF cold response pathway, but a CBF regulon that differs from that of freezing-tolerant *Arabidopsis*. Plant J..

[40] Phukan U.J., Jeena G.S., Tripathi V., Shukla R.K. (2017). Regulation of Apetala2/ethylene response factors in plants. Front. Plant Sci..

[41] Sun J., Peng X., Fan W., Tang M., Liu J., Shen S. (2014). Functional analysis of *BpDREB2* gene involved in salt and drought response from a woody plant *Broussonetia papyrifera*. Gene.

[42] Jangale B.L., Chaudhari R.S., Azeez A., Sane P.V., Sane A.P., Krishna B. (2019). Independent and combined abiotic stresses affect the physiology and expression patterns of *DREB* genes differently in stress-susceptible and resistant genotypes of banana. Physiol. Plant..

[43] Akbudak M.A., Filiz E., Kontbay K. (2018). DREB2 (dehydration-responsive element-binding protein 2) type transcription factor in sorghum (*Sorghum bicolor*): Genome-wide identification, characterization and expression profiles under cadmium and salt stresses. BioTech.

[44] Bihani P., Char B., Bhargava S. (2011). Transgenic expression of sorghum *DREB2* in rice improves tolerance and yield under water limitation. J. Agric. Sci..

[45] Shen Y.G., Zhang W.K., He S.J., Zhang J.S., Liu Q., Chen S.Y. (2003). An EREBP/AP2-type protein in *Triticum aestivum* was a DRE-binding transcription factor induced by cold, dehydration and ABA stress. Theor. Appl. Genet..

[46] Xue G.P., Loveridge C.W. (2004). HvDRF1 is involved in abscisic acid-mediated gene regulation in barley and produces two forms of AP2 transcriptional activators, interacting preferably with a CT-rich element. Plant J..

[47] Nakashima K., Ito Y., Yamaguchi-Shinozaki K. (2009). Transcriptional regulatory networks in response to abiotic stresses in Arabidopsis and grasses. Plant Physiol..

[48] Morimoto K., Mizoi J., Qin F., Kim J.S., Sato H., Osakabe Y., Shinozaki K., Yamaguchi-Shinozaki K. (2013). Stabilization of *Arabidopsis* DREB2A is required but not sufficient for the induction of target genes under conditions of stress. PLoS ONE.

[49] Liu S., Wang X., Wang H., Xin H., Yang X., Yan J., Li J., Tran L.S., Shinozaki K., Yamaguchi-Shinozaki K. (2013). Genome-wide analysis of *ZmDREB* genes and their association with natural variation in drought tolerance at seedling stage of *Zea mays* L.. PLoS Genet..

[50] Zhang J., Liao J., Ling Q., Xi Y., Qian Y. (2022). Genome-wide identification and expression profiling analysis of maize AP2/ERF superfamily genes reveal essential roles in abiotic stress tolerance. BMC Genom..

[51] Qin F., Kakimoto M., Sakuma Y., Maruyama K., Osakabe Y., Tran L.S., Shinozaki K., Yamaguchi-Shinozaki K. (2007). Regulation and functional analysis of ZmDREB2A in response to drought and heat stresses in *Zea mays* L.. Plant J..

[52] Zhao L., Wang P., Yan S., Gao F., Li H., Hou H., Zhang Q., Tan J., Li L. (2014). Promoter-associated histone acetylation is involved in the osmotic stress-induced transcriptional regulation of the maize *ZmDREB2A* gene. Physiol. Plant..

[53] Manna M., Thakur T., Chirom O., Mandlik R., Deshmukh R., Salvi P. (2021). Transcription factors as key molecular target to strengthen the drought stress tolerance in plants. Physiol. Plant..

[54] Ruelland V., Vaultier M.N., Zachowski A., Hurry V. (2009). Cold signalling and cold acclimation in plants. Adv. Bot. Res..

[55] Pearce R.S. (2001). Plant freezing and damage. Ann. Bot..

[56] Dias M.C., Oliveira H., Costa A., Santos C. (2014). Improving elms performance under drought stress: The pretreatment with abscisic acid. Environ. Exp. Bot..

[57] Mwando E., Angessa T.T., Han Y., Li C. (2020). Salinity tolerance in barley during germination- homologs and potential genes. J. Zhejiang Univ. Sci. B.

[58] Sakuma Y., Maruyama K., Osakabe Y., Qin F., Seki M., Shinozaki K., Yamaguchi-Shinozaki K. (2006). Functional analysis of an *Arabidopsis* transcription factor, DREB2A, involved in drought-responsive gene expression. Plant Cell.

[59] Sakuma Y., Maruyama K., Qin F., Osakabe Y., Shinozaki K., Yamaguchi-Shinozaki K. (2006). Dual function of an Arabidopsis transcription factor DREB2A in water-stress-responsive and heat-stress-responsive gene expression. Proc. Natl. Acad. Sci. USA.

[60] Reis R.R., da Cunha B.A., Martins P.K., Martins M.T., Alekcevetch J.C., Chalfun A., Andrade A.C., Ribeiro A.P., Qin F., Mizoi J. (2014). Induced over-expression of AtDREB2A CA improves drought tolerance in sugarcane. Plant Sci..

[61] Liu H., Lyu H.M., Zhu K., Van de Peer Y., Max Cheng Z.M. (2021). The emergence and evolution of intron-poor and intronless genes in intron-rich plant gene families. Plant J..

[62] Huang H., Xie S., Xiao Q., Wei B., Zheng L., Wang Y., Cao Y., Zhang X., Long T., Li Y. (2016). Sucrose and ABA regulate starch biosynthesis in maize through a novel transcription factor, ZmEREB156. Sci. Rep..

[63] Gao J., Shi J., Dong S., Liu P., Zhao B., Zhang J. (2018). Grain development and endogenous hormones in summer maize (*Zea mays* L.) submitted to different light conditions. Int. J. Biometeorol..

[64] Kumar S., Stecher G., Tamura K. (2016). MEGA7: Molecular evolutionary genetics analysis version 7.0. Mol. Biol. Evol..

[65] Barrs H.D., Weatherley P.E. (1962). A re-examination of the relative turgidity technique for estimating water deficits in leaves. Aust. J. Biol. Sci..

