# Supplementary material for: ZmDREB2.9 Gene in Maize (Zea mays L.): Genome-Wide Identification, Characterization, Expression, and Stress Response
Source: Plants (Basel). 2022 Nov 11;11(22):3060. doi: 10.3390/plants11223060 (PMC9694119; doi:10.3390/plants11223060)
Supplement: Supplementary file 1 [file plants-11-03060-s001.zip › plants-2006930-supplementary.pdf]

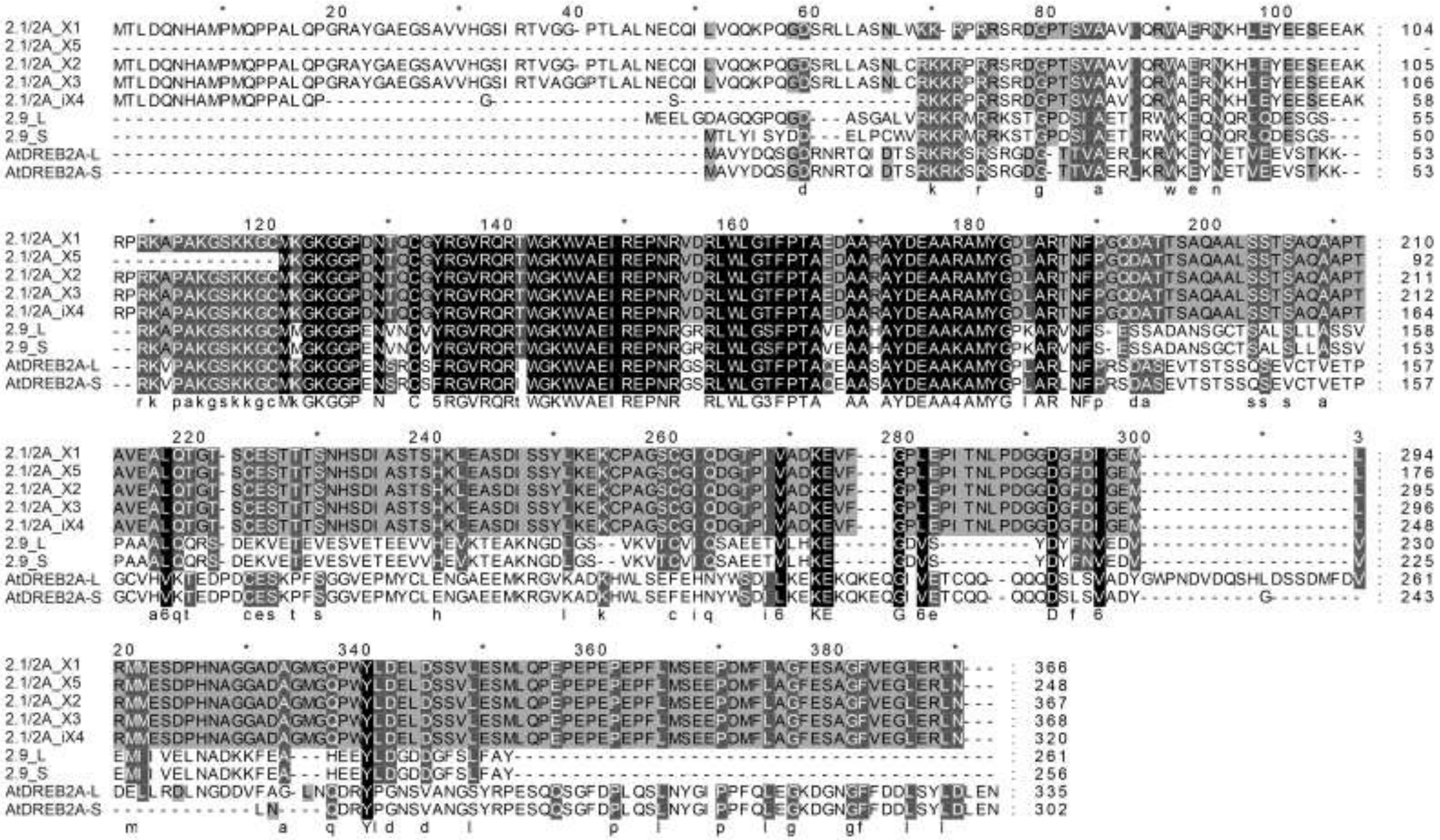

Figure S1. Alignment of ZmDREB2.1/2A, ZmDREB2.9 and AtDREB2A isoforms (indicated according to the Table 1).

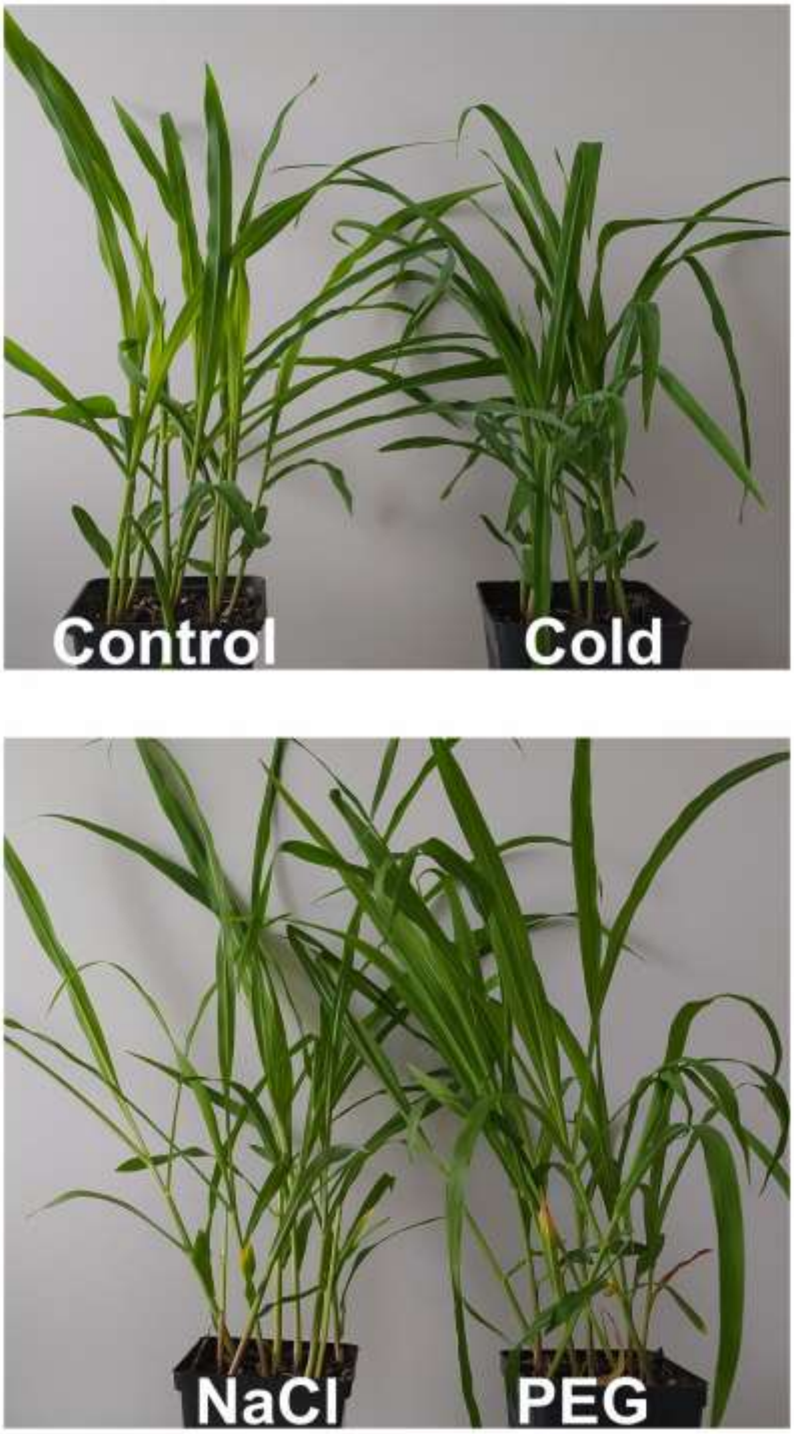

Figure S2. Photographs of maize seedlings 24 h after stresses (cold, salinity [NaCl], or drought [PEG]) compared to the untreated control. Seedlings after treatment with ABA are not shown, since outwardly, they did not differ from the control.

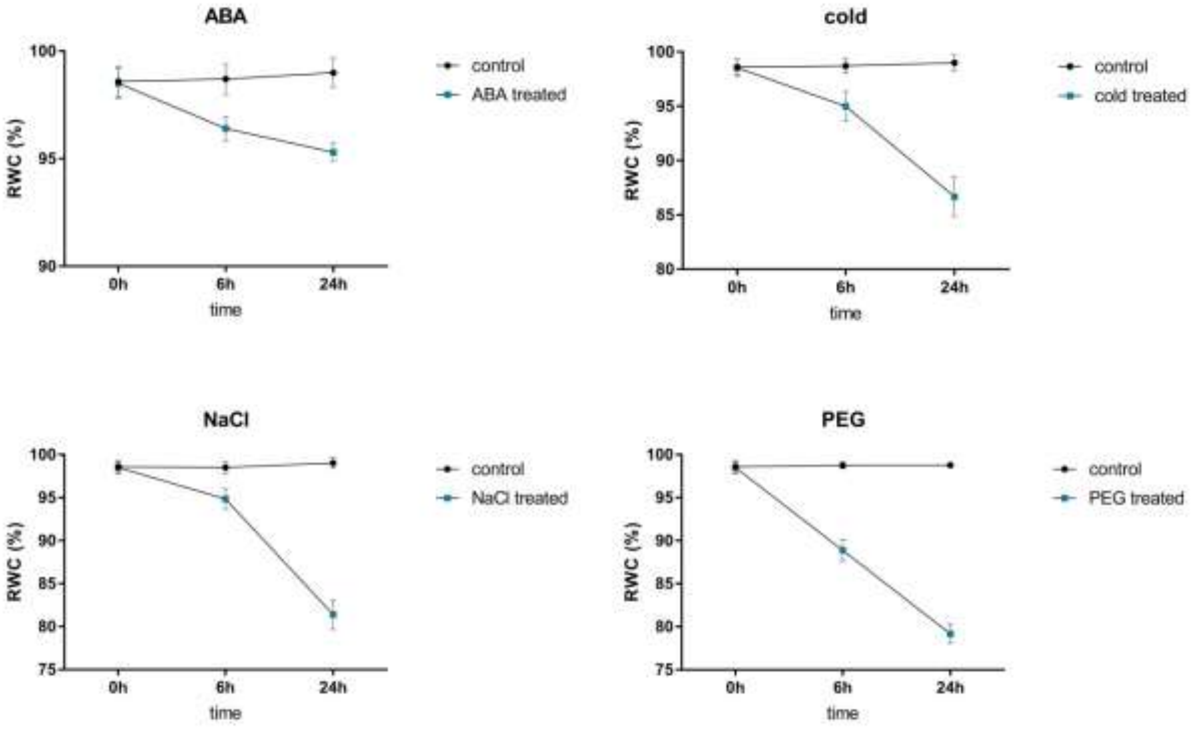

Figure S3. Relative water content in the leaf of corn seedlings 6 and 24 hours after stresses (ABA treatment, cold, salinity [NaCl], drought [PEG]) compared to the untreated control.

Table S1. List of primers for *ZmDREB2.1/2A–2.9* gene expression analysis.

Genes	Primer sequences (5′→3′)
ZmDREB2.1/2A (X2; X3)	GTTCCATCAGAACAGTAGCAGG<br>CAGATTAGAGGCCAGCAGTCG
ZmDREB2.1/2A (X1; iso1)	GTTCCATCAGAACAGTAGGAG<br>CAGATTAGAGGCCAGCAGTCG
ZmDREB2.2	GGACGACGACCCTCATGAC<br>GCCATCGAACCCCTCATGC
ZmDREB2.3	AAGAAGGGCTCCGGCTCGT<br>TACAGGAGCCGCAGGTAGG
ZmDREB2.4	CCTGGACCTGGACCTGGA<br>GAACTCCCTGAGGTCGAGC
ZmDREB2.5	CCCCAGATCGACCTCAGC<br>AGCTGGCTTTAGCAGTAGTCC
ZmDREB2.6	CAGGGAGTTCTTGCAACAGATC<br>GCCGCTGACGAAGCAAGACA
ZmDREB2.7	GATGTTCCAGACACCTGCACT<br>CCATCGCGGTGGTAGTGTC
ZmDREB2.8	GTGATGATGGCTGCCGTCC<br>TCCTGATGCAGAGGAGCCG
ZmDREB2.9-S (X1)	GACGATGAATTGCCCTGTTGG<br>CTCTTTCCACCACCTGATCG
ZmDREB2.9-L (iso1)	ATGGAGGAGCTGGGAGACG<br>CTCTTTCCACCACCTGATCG
